# Preclinical and Clinical Applications of Biomaterials in the Enhancement of Wound Healing in Oral Surgery: An Overview of the Available Reviews

**DOI:** 10.3390/pharmaceutics12111018

**Published:** 2020-10-24

**Authors:** Giacomo Picciolo, Matteo Peditto, Natasha Irrera, Giovanni Pallio, Domenica Altavilla, Mario Vaccaro, Giuseppe Picciolo, Alessandro Scarfone, Francesco Squadrito, Giacomo Oteri

**Affiliations:** 1 Department of Biomedical and Dental Sciences and Morphological and Functional Imaging, University of Messina, Via C. Valeria, 98125 Messina, Italy; giacomo.picciolo@unime.it (G.P.); matteo.peditto@unime.it (M.P.); daltavilla@unime.it (D.A.); giacomo.oteri@unime.it (G.O.); 2 Department of Clinical and Experimental Medicine, University of Messina, Via C. Valeria, 98125 Messina, Italy; nirrera@unime.it (N.I.); gpallio@unime.it (G.P.); vaccaro@unime.it (M.V.); alessandro.scarfone@unime.it (A.S.); 3 SunNutraPharma, Academic Spin-Off Company of the University of Messina, Via C. Valeria, 98125 Messina, Italy; beppepicciolo24@gmail.com

**Keywords:** biomaterials, oral wound healing, oral surgery

## Abstract

Oral surgery has undergone dramatic developments in recent years due to the use of biomaterials. The aim of the present review is to provide a general overview of the current biomaterials used in oral surgery and to comprehensively outline their impact on post-operative wound healing. A search in Medline was performed, including hand searching. Combinations of searching terms and several criteria were applied for study identification, selection, and inclusion. The literature was searched for reviews published up to July 2020. Reviews evaluating the clinical and histological effects of biomaterials on post-operative wound healing in oral surgical procedures were included. Review selection was performed by two independent reviewers. Disagreements were resolved by a third reviewer, and 41 reviews were included in the final selection. The selected papers covered a wide range of biomaterials such as stem cells, bone grafts, and growth factors. Bioengineering and biomaterials development represent one of the most promising perspectives for the future of oral surgery. In particular, stem cells and growth factors are polarizing the focus of this ever-evolving field, continuously improving standard surgical techniques, and granting access to new approaches.

## 1. Introduction

The birth of oral surgery is difficult to trace back. Considering exodontics as a part of this specialty, its roots may go back to the origins of dentistry itself. However, since the publication of Anselme Louis Bernard Berchillet’s “Traité des maladies et des opérations réellement chirurgicales de la bouche,” published in 1778, oral surgery has dramatically evolved and expanded together with the other surgical fields of medicine, effectively encompassing a wide range of procedures beyond exodontics, such as oral pathology, orthodontics, complex reconstructive techniques, and so on. These changes are intertwined with the continuous development of evidence-based medicine and new technologies that constantly challenge the very logic behind the surgical approach, from diagnosis to treatment. Improving oral surgery gives access to new and more complex clinical scenarios stemming from the increase in patients’ demands and age along with the increase in complications such as osteoradionecrosis, drug-related osteonecrosis, and peripheral trigeminal nerve injuries [1]. Bioengineering and the development of biomaterials have opened up one of the most promising doors to the future of oral surgery [2,3,4,5,6]; the ability to selectively and predictably regenerate tissue, such as bone, mucosa, and nerve tissue, using new technologies, is the key to improve the surgical outcomes of oral wound healing and simplify the current reconstructive approach to complex cases, such as the atrophic mandible or soft tissue management in periodontal patients. On the way to reach this goal, clinicians are witnessing a significant shift in the field of biomimetic biomaterials and bioactive ones, specifically engineered to promote wound healing exploiting molecules or additives with different purposes, such as targeted cellular recruitment or antimicrobial potential, or specific design elements, shifting from simple scaffolds to matrixes providing a spatial orientation for tissue growth [7]. The aim of this review is to provide a comprehensive overview of the current knowledge on biomaterials used in oral surgery and to review the available systematic reviews concerning biomaterials used in oral surgery and their impact on post-operative wound healing.

## 2. Methods

### 2.1. Development of a Protocol

The overview has been carried out in agreement with a defined protocol, that included: the identification of a focused question, search strategy, criteria for review selection and inclusion.

### 2.2. Defining the Focused Question

The focused question was defined as: ‘What is the clinical and histological effect of biomaterials as adjuvants of the oral surgical procedures on oral wound healing as evaluated by systematic literature reviews?’

### 2.3. Search Strategy

**Figure 1:**
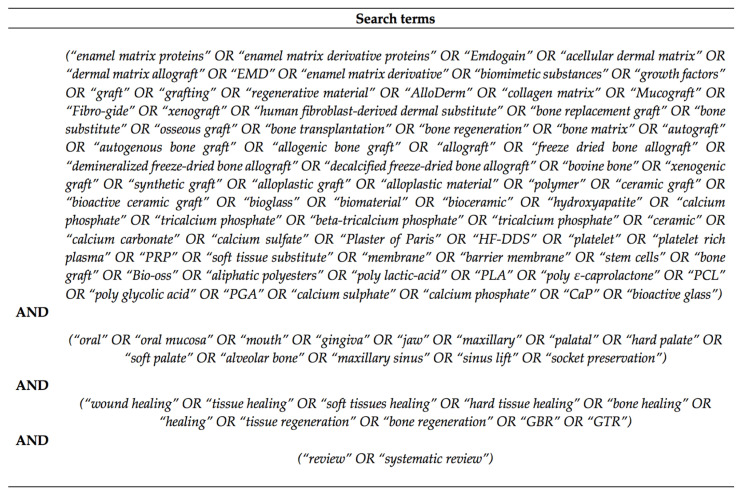
Search strategy.

The literature was searched for reviews published up to July 2020, using the MEDLINE database. Different combinations of search terms were developed to identify suitable studies. Papers from reference lists of the considered reviews articles were also manually scanned and screened. Search strategy is shown in Figure 1.

### 2.4. Criteria for Review Selection and Inclusion

The review selection was performed by two independent reviewers. Disagreements were resolved by a third reviewer. Selection was limited to reviews that included clinical studies describing both clinical and histological effects of biomaterials on post-operative wound healing in oral surgical procedures. Reviews analyzing non-surgical oral procedures (e.g., orthodontics and conservative dentistry therapies) were discarded. A time limitation of a minimum of 6 weeks for the postoperative evaluation period was applied.

## 3. Results

A total of 41 articles were included in the final review. Final papers are summarized in Table 1, while excluded articles are specified in Table 2.

Results are discussed below and divided into specific topics: stems cells, bone grafts, and growth factors.

### 3.1. Stem Cells

#### 3.1.1. Background

Stem cells can be defined as undifferentiated cells characterized by a set of unique properties. A stem cell is capable of proliferation, self-renewal, production of differentiated daughter cells, self-maintenance of their population, and regeneration of injured tissue. An additional key aspect behind stem cells behavior is the flexibility in their behavior based on environmental conditions [58,59]. Stem cells belong to two main subtypes: pluripotent (or totipotent), able to differentiate in any kind of human cell, and multipotent, that can develop into multiple cell types within their lineage. They can be successfully isolated from the inner part of the blastocyst, prior to the implantation of the embryo, together with fetal and adult tissue. Adult stem cells are generally multipotent and are found in most human tissues, as they support the active cell turnover for tissues undergoing self-renovation at different degrees, and enable tissue repair by replacing damaged or lost cells. Adult stem cells can be harvested from various tissues, such as the bone marrow and the oral cavity. In fact, it is possible to find multipotent adult stem cells in exfoliated deciduous teeth, dental pulp, and periodontal ligaments that show osteogenic and neurogenic capacities [60]. Mesenchymal stem cells obtained from the bone marrow are able to differentiate into various cell types and can respond to the medium where they are inserted to differentiate themselves in the appropriate tissues, as needed [6]. Donor area of choice is usually the iliac crest bone marrow; however, the harvesting procedure from this site may be trivial and painful, so new solutions are being looked for to bypass this kind of inconvenience. Interestingly, mandible periosteum and maxillary tuberosity have been proved as a reliable source of mesenchymal stem cells with osteogenic potential, easy to access under local anesthesia and with low to no post-operative discomfort [61].

#### 3.1.2. Overview of Reviews

In total, six reviews were found on the topic, one considering only animal studies, three only human study, and two accounting for both animal and human studies. The review from Amghar-Maach et al. [8], focused on animal studies, assesses the efficacy of dental pulp stem cells (DPSC) in the regeneration of periodontal defects, but remarks how the biomaterial architecture is relevant to the regeneration outcome. In fact, grafting stem cells in the form of cell sheets leads to better results when compared to the injection of dissociated cells. Additionally, pairing stem cells with growth factor such as hepatocyte growth factor (HGF) may favor DPSC differentiation. Correia et al. [16] and Mangano et al. [30] discuss the impact of mesenchymal stem cells in maxillary sinus augmentation, even if paired up with other biomaterials. Mesenchymal stem cells (MSCs) show a positive impact on wound healing and bone regeneration considering vital bone and vital bone percentage, leading to better outcomes in terms of osteogenesis and bone volume gain. Socket preservation procedures, analyzed by Pranskunas et al. [36], show an increase in the clinical and radiographical aspects of wound healing in both animal and human studies; however, no significant difference from a histological point of view was found. Considering implant-related bone regeneration procedures [45] (Varshney et al.), both adipose-derived and bone marrow-derived stem cells have been proved to improve the expected results. However, while this efficacy is particularly relevant in animal models, the treatment of large defects in humans does not always relate to a predictable outcome in terms of regeneration. Therefore, even if very promising, stem cell use in the improvement of oral wound healing is not highly predictable. A better understanding of cellular interactions in the healing phases could help overcome this flaw. The combined use of stem cells and growth factors may improve the efficacy of the regenerative approach in a significant way.

### 3.2. Bone Graft and Resorbable Membranes

#### 3.2.1. Background

Bone grafts are natural or synthetic biomaterials used in the regeneration of defective bone volumes. They can be classified according to their source, microscopic architecture, form, and blood supply [62].

Considering their origin, bone grafts can be defined as:

- Autografts, obtained from the same individual that receives the graft;
- Isografts, from an individual from the same species sharing the same antigenic profile (twins);
- Allografts, harvested from an individual from the same species but with a different antigenic profile;
- Xenografts. obtained from species other than human;
- Alloplastic materials, synthetic bone graft substitutes [63,64].

Moreover, bone grafts can exhibit different properties that provide the rationale for their use in regenerative procedures:

- Osteogenesis: the graft contains living osteoblasts that contribute to new bone formation;
- Osteoinduction: the graft is able to stimulate the differentiation of osteoprogenitor cells into osteoblasts;
- Osteoconduction: the graft acts as a scaffold to sustain the development of capillaries and precursor bone cells [65].

Osteogenesis requires the presence of mesenchymal cells able to differentiate into mature osteoblasts (such in autografts). Osteoinduction usually relies on the presence of growth factors, molecules able to mediate cells recruitment, proliferation, and differentiation, and represents one of the most challenging tasks for the development of bone graft substitutes [66].

Regardless of their osteogenetic and osteoinductive properties, every bone graft has to grant a three-dimensional mechanical structure that hosts and supports cells and extra-cellular matrix [62]. The key feature to a scaffold is the porosity of its structure, since pores increase contact surface of the bone graft, favoring its degradation, and allow cell migration and proliferation [30]. Pore diameter, together with pore morphology and interconnectivity, [67] seem to affect cell behavior, favoring neoangiogenesis with a diameter greater than 300 µm, and osteoblasts migration, adhesion, and proliferation with a diameter of 200–400 µm. Current literature suggests that a porosity of more than 50% by volume and pore sizes of 200–800 µm are the most adequate feature for the development of bone tissue [62,68,69]. These grafting properties are defined as osteocondustive, as described above.

The ideal bone graft should exhibit osteogenic, osteoinductive, and osteoconductive properties while lacking antigenic, teratogenic, or carcinogenic reactions, favor neoangiogenesis, be resorbable, possess a hydrophilic nature, and have low morbidity and cost. Resorbable membranes are devices commonly paired up with bone grafting materials; among them, resorbable collagen membranes (RCMs) are the most commonly found in clinical practices [70]. RCMs are manufactured from allogeneic or xenogeneic sources to manage oral wounds such as extraction sockets, sinus-lift, and ridge augmentation procedures, and periodontal and endodontic surgeries [71,72,73,74]. They are one of the essential tools in guided bone regeneration (GBR) techniques, enhancing wound healing through promotion platelet aggregation, clot stabilization, and fibroblast attraction [75,76]. Time of resorption varies from 2 to 32 weeks and they are biocompatible, easy to manipulate, and with low immunogenicity [77]. They are available as membranes, plugs, or pads for ease of use [70].

#### 3.2.2. Bone Grafts Categories

##### Autografts

Autografts are often regarded as the ‘gold standard’ among bone grafts due to their osteogenic and osteoinductive properties. Additionally, they are almost safe from the risk of immune reaction/rejection, being harvested from the same subject that receives the graft itself. However, one of their major drawbacks is represented by the necessity of a surgical intervention to collect the graft, and this may affect a patient’s systemic health, increase morbidity, and expose the subject to the risk of chronic postoperative pain and hypersensitivity of the donor area [78]. In oral surgery, autografts are proven to not be able to counteract the volume contraction of the hard tissues of the edentulous sites [79]. Autograft can be harvested by various intra- and extra-oral donor sites. Intraoral donor sites include edentulous ridges, extraction sockets, mandibular ramus, symphysis, and maxillary tuberosity, while extraoral donor sites are tibia, iliac crest, and calvarium [80,81,82,83,84,85,86,87,88,89,90,91,92,93,94,95].

##### Allografts

Allografts are collected from individuals, either dead or alive, of the same species but with a different genotype, processed in order to prevent the host’s immune response and transmission of infectious diseases [89]. They are available as cortical, cancellous, or cortico-cancellous grafts, in various shapes and sizes. Allografts processing has evolved over the years, from the use of fresh frozen bone, simply frozen at −80 °C and no longer used due to the risk of disease transmission and immune response, to the demineralized freeze–dried bone allograft (DFDBA), processed to preserve the organic part that contains bone morphogenetic proteins, growth factors responsible for the graft osteoinductive properties [96].

##### Xenografts

Xenografts are obtained from donors of a species other than the host’s one, and mostly act as scaffolds showing osteoconductive features and slow resorption time. They can be used both alone and paired up with growth factors or other grafts to enhance their properties. Their lack of osteogenetic and osteoinductive properties is balanced by their availability and relatively low cost. Originating from non-human species, the risk for disease transmission and immunogenicity has to be accounted when using xenografts [97].

Among the many available xenografts, the main categories refer to bovine substitutes, equine substitutes, porcine substitutes, algae substitutes, and coral substitutes.

##### Alloplastic Materials

Alloplastic materials are biomimetic synthetic bone substitutes, characterized exclusively by osteoconductive features, with no osteoinductive or osteogenic properties. Therefore, they act as a scaffold to support cell migration, proliferation, growth and bone tissue formation [98]. Considering the many chemical and physical properties, they are considered the most heterogeneous group of materials that includes, among the many synthetic bone substitutes, calcium phosphate, calcium carbonate, calcium sulfate, bioactive glasses, and polymers.

#### 3.2.3. Overview of Reviews

A total of twenty-five reviews were found regarding bone grafts and different surgical procedures, such as alveolar socket/ridge preservation, periodontal regeneration, atrophic jaws augmentation, sinus augmentation procedures, and alveolar ridge splitting/espansion technique (ARST).

Preservation procedures found controversial results in literature, with not always clear evidence regarding new bone formation when comparing natural healing to grafts [10,19,31,48].

Magnesium-enriched hydroxyapatite (mHA), calcium sulphate, and porcine grafts granted a better outcome when compared to natural healing, while DFDBA proved to be the most efficient allograft among all [10].

Short term bone wound healing, from 3 to 4 months from dental extraction, shows similar clinical, radiographical, and histological characteristics regardless of the use of a bone graft.

Chan et al. [14] found conflicting results concerning the percentage variation of the vital bone with the use of xenografts, ranging from –22% (decrease) to 9.8% (increase), while connective tissue formation likely decreases with the use of bone substitute. Only limited evidences support an increase in vital bone formation following the use of alloplasts. Significant amounts of hydroxyapatite and xenograft particles (15 to 36%) were found at the healing site at an average of 5.6 months after grafting, as a proof of their stability and resistance to resorption. According to Horvath et al. [25], only a limited cluster of studies report a statistically significant increase in trabecular bone formation when using bone grafts in the alveolar ridge preservation; the use of bone substitute does not prevent ridge resorption but rather delays it, due to the permanence of graft particles inside the healing sockets. From the histological point of view, conflicting evidences are found regarding the benefits of ridge preservation, with no active promotion of bone formation sustained by bone grafts, and rather peculiar histological pictures of what resembles a foreign-body reaction from the host to the bone substitute particles [19,34].

While showing an impact on the reduction in the vertical bone dimension following tooth extraction, socket grafting showed no clear evidence of bone dimensional preservation, bone formation, or keratinised tissue dimensions [29].

On the other hand, Willenbacher et al. [46] found an increase in the preserved bone quota, approximately 1.31 to 1.54 mm bucco-oral bone width and 0.91 to 1.12 mm bone height, in alveolar sockets preserved with grafting materials.

The use of alternative graft solutions, such as tooth-bone graft, demonstrated no added benefits over conventional graft materials [22].

Allogeneic bone blocks represent a good alternative to autologous bone blocks, however, histological analysis highlights differences in their behavior during the healing phases. At 6 months. no connective tissue was found and the presence of inflammatory cells was meaningfully lower when recurring to autologous bone, while in the allogeneic blocks large segments of necrotic bone with empty osteocytes lacunae and little osteoclastic activity were found, along with blood vessels invading the Haversian canals of the graft [33].

The use of bone blocks enables vertical-deficient sites to be rehabilitated with implants in animal models [40].

Advanced atrophic bone augmentation techniques, such as ARST, may benefit from the use of bone grafting to preserve buccal bone height and width [11].

Autologous bone shows the best results in sinus augmentation procedures despite its high resorption rate (40%) in animal studies. This downside can be overcome by mixing it to other bone grafts, such as porous hydroxyapatite or bioglasses [12,15,26].

Periodontal regeneration of intrabony defect using bone grafts proved to be superior in terms of regenerative outcomes when compared to simple flap surgery with no use of biomaterials in both animal and human studies, with autologous bone showing the most favorable results [27,34,47].

The use of grafts combined with membranes proved the best result in terms of periodontal regeneration [37], especially in supra-alveolar and two wall intrabony defects models in animals. Three wall intrabony defects do not benefit consistently of the use of grafts or membrane systems [41].

Regeneration of gingival defects is, however, according to Danesh-Meyer et al. [17], compromised by the wound stabilizing effect of the membrane itself, which does not provide adequate space to promote periodontal regenerational while simultaneously impeding apical migration of the gingival epithelium.

Interestingly, alloplastic grafts likely support periodontal repair rather than regeneration [38] and appear to show limited amounts of periodontal regeneration when compared to the other biomaterials [42].

Overall, bone grafts and membranes represent an essential tool in the hard and soft tissue regeneration. In recent years, the efficacy of bone grafts in the socket/ridge preservation techniques has been debated and more and more controversial evidences are emerging, while technology evolves and opens up new scenarios, such as in the case of Computer-Aided Design/Computer-Aided Manufacturing (CAD/CAM) manufactured bone scaffolds. The key to an optimal wound healing relies, as always in the choice of the correct device, in a deep comprehension of their biological and mechanical properties.

### 3.3. Growth Factors

#### 3.3.1. Background

Growth factors (GFs) are molecules able to regulate DNA synthesis, chemotaxis, matrix synthesis, and promote cellular growth, proliferation, and cellular differentiation, usually of proteic or steroid nature.

Among the wide category of growth factors, some have polarized the research attention over the recent years, such as:

- Bone morphogenic proteins (BMPs): cytokines able to stimulate bone cell differentiation and promote new bone formation, responsible for the osteoinductive features of bone grafts;
- Fibroblast growth factor (FGF) and vascular endothelial growth factors (VEGF): mostly stimulate neoangiogenesis;
- Platelet-derived growth factor (PDGF): known as one of the initiators of wound healing, with multiple functions ranging from chemotaxis and mitogenesis to promotion of angiogenesis, acts on both soft and hard tissues;
- Transforming growth factor beta (TGF-β) and insulin-like growth factor (IGF): regulate collagen and fibronectin synthesis through osteoblasts or fibroblasts stimulation;
- Amelogenins: extracellular matrix proteins secreted by ameloblasts that regulate hydroxyapatite crystal growth and orientation and are able to promote periodontal tissues regeneration; in the clinical practice, they are commonly found in enamel matrix derivatives (EMD) compounds, a mix of enamel matrix proteins (EMP), of which amelogenins represent circa 90% of the total protein quota [99,100];
- Statins: recently discovered to possess anti-inflammatory, antimicrobial and pro-osteogenic properties.

An important biological vehicle responsible for delivering GFs to wounded sites is represented by platelets that, in addition to their procoagulant effect, release many biomolecules like PDGF, TGF-β, VEGF, etc.

Therefore, despite the availability of recombinant GFs, the use of autologous platelet concentrates has found many applications in oral surgery.

Platelet concentrates belong to four main categories:

1. Pure Platelet Rich Plasma (P-PRP) or leukocyte-poor PRP that does not contain leukocytes;
2. Leukocyte and PRP (L-PRP) products;
3. Pure Platelet Rich Fibrin (P-PRF) or leukocyte-poor PRF;
4. Leukocyte and PRF (L-PRF).

PRP is a first-generation platelet concentrate containing platelets in super-natural concentration and minimal amount of natural fibrinogen. Platelets’ α granules are responsible for the release of growth factors within 3–5 days of platelet activation, which sustain their stimulation of proliferative phase for 10 days after release. However, calcium chloride and bovine thrombin are added to reach gel consistency and these components may interfere with wound healing [101,102].

Preparation rich in growth factors (PRGF-Endoret) technology was invented as an answer to some of the limitations of PRP preparations. The clot activator, calcium chloride, leads to the formation of native thrombin. This mimicked physiological clotting process enables a more sustained release of growth factors. Moreover, this procedure reduces the risk of immunological reactions and disease transmission associated with the use of exogenous bovine thrombin [103].

PRF represents a new generation platelet concentrate, an evolution of PRP. Similar to the blood clot, it is a tetramolecular fibrin matrix that contains all the molecular and cellular elements, such as platelets, leukocytes, cytokines, and circulating stem cells, that promote healing simultaneously being more stable and homogenous. Furthermore, 20 PRF does not require addition of bovine thrombine or other substances, thus it does not share the coagulant-related drawbacks of PRP [104,105].

#### 3.3.2. Overview of Reviews

A total of thirteen reviews were considered regarding the use of growth, both autologous or recombinant.

In sinus augmentation, PRP does not significantly affect the histological density and quality of the regenerated bone; however, early wound healing was observed [9].

According to Stähli et al. [43], while having no evidence supporting the clinical benefit of PRP in healthy patients, PRP might have a positive effect on wound healing and bone regeneration in compromised patients.

PRF, however, showed superior outcomes in bone regeneration procedures, as per ridge dimension, bone regeneration, osseointegration process, and soft tissue healing [44].

According to Darby and Morris [18], periodontal regeneration performed through the use of PDGF led to greater CAL gain of around 1mm, a greater percentage bone fill of around 40%, and an increased rate of bone growth, compared to an osteoconductive control (β-TCP), with no particular adverse effects. This consideration is backed up by the systematic review of Giannobile and Somerman [23], assessing that PDGF promotes periodontal regeneration at the histological level.

The efficacy of PDGF may be further improved, associating it with an osteoconductive scaffold matrix [35].

Similarly, EMD were found to consistently promote CAL gain and probing depth reduction when compared to flap surgery alone, and its effect is improved using it in combination with graft materials [23,28].

The efficacy of EMD is further highlighted in the treatment of gingival recession [16], improving soft tissue height and thickness; using EMD together with coronally advanced flaps in root coverage seemingly leads to periodontal regeneration with formation of root cementum, periodontal ligament, and alveolar bone [32]. Platelet concentrates showed positive effects on the healing outcome of both soft and hard tissue in the post-extraction alveolar socket, with a significant increase in the keratinized mucosa quota and in the new bone formation percentage, although this result is controversial [20,21].

Local application of statins shows an apparent osteogenic and angiogenic effect in periodontal defects models in animal studies; topical simvastatin enhances wound healing and improves patient outcomes, stimulating bone formation, promoting soft tissue healing, as well as reducing post-operative pain and inflammation [24,39].

## 4. Conclusions

The development of biomaterials represents one of the most promising perspectives for the future of oral surgery. In particular, stem cells and growth factors are polarizing the focus of this ever-evolving field, continuously improving standard surgical techniques, and granting access to new approaches. Bone grafts and membranes usually play a pivotal role in GBR procedures. Despite their long history as essential tools in regenerative procedures, controversial evidences are emerging regarding the socket/ridge preservation techniques, that represent the basic approach in the modern oral surgery to the post-extraction socket and edentulous ridge. Technology evolves and opens up new scenarios, such as in the case of CAD/CAM manufactured bone scaffolds.

The regenerative properties of the biomaterials used in oral surgery may be improved thanks to growth factors. Their combined use, in fact, likely enhances the healing processes and favors early wound healing after oral surgical procedures.

## Figures and Tables

**Table 1:** Included articles.

Authors	Reference	Year	Included Studies	Biomaterial Type	Wound Healing Type	Principal Findings
Amghar-Maach et al.	[8]	2019	5 (animal studies)	stem cells	regeneration of periodontal bone	More bone volume/better periodontal health achieved with dental pulp stem cells (DPSC)grafted as cell sheets. Addition of hepatocyte growth factor (HGF) favors DPSC differentiation.
Arora et al.	[9]	2010	6 (randomized clinical trials—RCT human studies)	growth factors (PRP)	sinus augmentation procedures	No statistically significant difference was observed in histological bone density and quality of the regenerated bone.
Barallat et al.	[10]	2014	34 (human studies)	bone grafts	ridge preservation procedures	Controversial impact of grafting on preservation. Calcium sulfate, porcine xenograft, and magnesium enriched hydroxyapatite (MHA) showed a statistically significant additional benefit when compared with healing by blood clot formation. Demineralized freeze-dried bone allograft (DFDBA) seems to be more efficient than freeze-dried bone allograft (FDBA) in terms of percentage of newly formed bone.
Bassetti et al.	[11]	2015	24 (18 human and 6 animal studies)	bone grafts	alveolar ridge splitting/expansion technique (ARST)	ARST seems to be a well-functioning one-stage alternative to prolonged two-stage horizontal grafting procedures.
Browaeys et al.	[12]	2007	26 (animal studies)	bone grafts	sinus augmentation procedures	Autogenous bone represents the gold standard for sinus augmentation procedures and shows better performances with BBM or porous hydroxyapatite. Homogenous Demineralized Freeze-Dried Bone (DFDB) provides better results than heterogenous DFDB.
Chambrone and Tatakis	[13]	2015	234 (not specificated)	bone grafts and EMD	periodontal regeneration	All root coverage (RC) procedures can offer significant drop in recession depth and clinical attachment level (CAL) improvement for Miller class I and II recession-type defects. subepithelial connective tissue graft-based procedures offered the best results for clinical practice because of their greater percentages of mean and complete RC, together with significant increase in keratinized tissue.
Chan et al.	[14]	2013	8 (human clinical trials)	bone grafts	socket preservation procedures	Conflicting results were found with the use of xenografts on changes in the percentage of vital bone. Partial evidence suggested that alloplasts rise the amount of vital bone formation. Higher % of connective tissue was significantly reduced with the use of bone substitutes. Significant quantities of hydroxyapatite and xenograft particles detected in the healed sockets at an average of 5.6 months after grafting.
Corbella et al.	[15]	2015	84 (human studies)	bone grafts	lateral sinus floor elevation procedure	Autogenous bone, bovine bone, and a mixture of tricalcium phosphate (TCP) and hydroxyapatite (HA) show predictable results.
Correia et al.	[16]	2017	18 (11 clinical trials—CT and 7 RCT)	bone grafts and stem cells	sinus lift procedures	Only a few studies have demonstrated potential of regenerative medicine in sinus lift. After 6 months significative differences were outline in the vital bone and percentage of residual graft, while results from the connective tissue were not. Same conclusions were described with the use of periosteum-derived cells with collagen matrix
Danesh-Meyer	[17]	2001	30 (human studies)	bone grafts and membrane	guided tissue regeneration	Barrier membranes do not show to provide adequate space to predictably support periodontal regeneration in gingival recession defects.
Darby and Morris	[18]	2013	5 (human studies)	growth factors (PDGF-BB)	periodontal regeneration	The use of rhPDGF-BB led to greater CAL gain, augmented rate of bone growth, and greater percentage bone fill related to an osseoconductive control.
De Risi et al.	[19]	2013	38 (human studies)	bone grafts	ridge preservation procedures	No major histomorphometrical and histological differences arose among different procedures or when compared to spontaneous healing.
Del Fabbro et al.	[20]	2017	33 (human studies)	growth factors (APC)	socket preservation procedures	Soft tissue healing was statistically better for sockets treated with Autologous Platelet Concentrates (APC) seven days after surgical procedures. New bone was statistically greater for APC group in one study.
Del Fabbro et al.	[21]	2018	8 (human studies)	growth factors (plasma-rich growth factors—PRGF)	alveolar socket healing	Better and faster epithelialization was observed in the sites treated with PRGF. The measurement of the thickness of the epithelial layer resulted in a thicker layer in the sockets treated with the PRGF.
Gharpure and Bhatavadekar	[22]	2017	26 (18 animal studies and 8 human studies)	bone grafts	bone regeneration	Histological examination of the grafted sites from several studies revealed the generation of a dentin-bone complex, where tooth-bone graft was enclosed by newly forming bone. All reports failed to show complete resorption of the graft material and its substitution by newly formed bone.
Giannobile and Somerman	[23]	2003	60 (human and animal studies)	growth factors (Enamel matrix derivative—EMD, bone morphogenetic proteins—BMP)	periodontal wound healing	EMD promotes bone regeneration and CAL gain. PDGF-BB promotes periodontal regeneration at the histologic level.
Gupta et al.	[24]	2019	16 (10 animal studies and 6 human studies)	statins	oral wound healing	The topical application of simvastatin and chitosan gel could be used as a novel therapeutic approach o improve healing and reduced pain in the palatal donor site following the free gingival grafts (FGG) procedure.
Horváth et al.	[25]	2012	14 (human studies)	bone grafts	ridge preservation procedures	Conflicting evidence exists on the benefit of alveolar ridge preservation (ARP) at the histological level. ARP does not appear to stimulate de novo hard tissue formation routinely. In addition, some graft materials may interfere with healing.
Ioannou et al.	[26]	2014	5 (human studies)	bone graft (bioactive glass)	bone regeneration	The combination of bone graft with autogenous bone chips in a 1:1 ratio is a successful treatment modality for the direct sinus augmentation, with histological results comparable to 100% autogenous bone.
Ivanovic et al.	[27]	2014	45 (animal studies)	bone grafts	periodontal regeneration	Among the used biomaterials, autografts, in combination with flap surgery, shown the most favorable outcomes, whereas the use of most biologic factors revealed inferior results compared to flap surgery
Kao et al.	[28]	2015	124 (human studies)	growth factors (EMD, NBM, PRP, NHA)	periodontal regeneration	Histologic evidence of periodontal regeneration has been demonstrated when EMD is used in conjunction with nanocrystalline hydroxyapatite (NHA), autogenous bone, a bovine-derived natural bone mineral (NBM), bioactive glass, NBM + PRP,) or biphasic calcium phosphate. The most part of the studies indicate no added benefits in either radiographic or clinical gains when EMD is used with the addition of graft materials.
MacBeth et al.	[29]	2016	9 (human studies) for q1 and 37 (human studies) for q2	bone grafts	ridge preservation procedures	Debated data are available on alterations of width of the keratinised tissue following GBR.
Mangano et al.	[30]	2015	39 (21 human and 18 animal studies)	stem cells	sinus augmentation procedures	BMSCs + PRP compose could give better outcomes in bone volume gain and osteogenesis comparable to that achieved by particulate cancellous bone in MSA.
Maroulakos et al.	[31]	2018	43 (6 human and 37 animal studies)	scaffold materials and grafts	bone regeneration	The immediate and long-term bone repair was considered successful for the time of observation by biochemical, histological, micro-computed tomographic or histomorphometric findings
Miron et al.	[32]	2016	19 (human and animal studies)	growth factors EMD)	periodontal regeneration	The use of EMD for the management of gingival recessions utilized alone is capable of improving regeneration and enhance soft tissue height/thickness. Application of EMD during conjunction with Coronally Advanced Flap (CAF) resulted in increased formation of alveolar bone, root cementum and periodontal ligament
Monje et al.	[33]	2014	15 (human studies)	bone grafts	maxillary augmentation	Histologic analysis revealed that allogeneic block grafts perform differently in the early stages of healing when compared to autogenous block grafts
Murphy and Gunsolley	[34]	2003	89 (human studies)	bone grafts	periodontal regeneration	Augmentation materials procedures, in addition to the physical barrier, enhance the regenerative outcome in the treatment of furcation defects treated with GTR. On the other hand, in the treatment of intrabony defects, there is no advantage to the use of augmentation materials in addition to the use of the physical barrier
Tavelli et al.	[35]	2020	63 (human studies)	growth factors (PRGF)	periodontal regeneration	There is strong evidence that recombinant human platelet- derived growth factor (rhPDGF) is efficient in the regeneration of intrabony defects when applicated in combination with a bone matrix. In particular, rhPDGF benefits from the delivery with an osteoconductive scaffold matrix. Clinical and histological results confirmed that rhPDGF in combination with a scaffold was also efficient in the treatment of furcation defects.
Pranskunas et al.	[36]	2019	11 (9 human and 2 animal studies)	stem cells	socket preservation procedures	The use of bioactive osteogenic molecules or mesenchymal stem cells supports bone regeneration after tooth extraction. Histologically, no particular differences are revealed between test and control groups.
Reddy et al.	[37]	2015	N.S.	bone grafts	periodontal regeneration	Histologic proof of periodontal regeneration after the application of a regenerative treatment for the management of maxillary distal, mesial, facial and mandibular lingual or facial Class II furcation defects has been demonstrated in several studies. Evidence of histologic periodontal regeneration in mandibular Class III defects is limited to one case report. Favorable results after a regenerative therapy for maxillary Class III furcation defects are limited to few clinical case reports. In Class I furcation defects, regenerative therapy could be useful in some clinical scenarios, although generally Class I furcation defects may be treated with non-regenerative therapies.
Reynolds et al.	[38]	2003	49 (human studies)	bone grafts	regeneration of periodontal bone defects	Demineralized freeze-dried bone allograft (DFDBA) boosts the production of a new attachment apparatus in intrabony defects, while open flap debridement (OFD) results in periodontal repair characterized principally by the formation of a long junctional epithelial attachment. Several observational studies provide a large numbers of histological evidences that autogenous and demineralized allogeneic bone grafts increase the formation of new attachment. Few data also suggest that xenogenic bone grafts can sustenance the formation of a new attachment apparatus
Roca-Millan et al.	[39]	2019	15 (animal studies)	statins	Regeneration of Periodontal bone defects	Bovine bone, autogenous bone and a mixture of tricalcium phosphate (TCP) and hydroxyapatite (HA) show predictable results.
Rocchietta et al.	[40]	2008	18 (animal and human studies)	bone grafts	bone regeneration	Only a few studies have established the potential role of regenerative medicine in sinus lift. Statistical significance differences were outlined after 6 months in the vital bone and percentage of residual graft, while outcomes from the connective tissue were not. Same conclusions were described with the use of periosteum-derived cells with collagen matrix.
Sculean et al.	[41]	2008	10 (Animal Studies)	Bone Grafts	Periodontal regeneration	Barrier membranes do not provide adequate space to predictably support periodontal regeneration in gingival recession defects.
Sculean et al.	[42]	2015	58 (Human Studies)	Bone Grafts	Periodontal regeneration	The use of rhPDGF-BB led to greater percentage bone fill, greater CAL gain and increased rate of bone growth compared to an osseoconductive control.
Stähli et al.	[43]	2018	22 (15 RCT and 7 CCT)	Growth Factors (PRP)	Bone Regeneration	No major histomorphometrical and histological differences occurred among different techniques or when compared to spontaneous healing.
Strauss et al.	[44]	2018	12 (RCT)	Growth Factors (PRP)	Bone Regeneration	Faster and better epithelialization was appreciated in the sites treated with PRGF. The measurement of the thickness of the epithelial layer resulted in a thicker layer in the sockets treated with the PRGF.
Varshney et al.	[45]	2020	10 (Human Studies)	Stem Cells	Bone Regeneration	Soft tissue healing was statistically better for sockets treated with Autologous Platelet Concentrates (APCs) seven days after surgery procedures. New bone was statistically greater for APC group in one study.
Willenbacher et al.	[46]	2015	18 (Human Studies)	Bone Grafts	Ridge Preservation Procedures	Histological examination of the grafted sites from a large number of papers showed the formation of a dentin-bone complex, where tooth-bone graft was enclosed by newly forming bone. All papers failed to demonstrate a complete resorption of the graft material and its substitution by newly formed bone.
Yen et al.	[47]	2013	22 (Human and Animal Studies)	Bone Grafts	Guided Tissue Regeneration (GTR)	Despite the quality assessments is different between human and animal studies, some papers suggested that animal models and human results displayed comparable bone-filling ratios in infrabony defects treated with GTR only or with GTR + bone grafting.
Zhao H. and Zhao L.	[48]	2020	5 (Human Studies)	Bone Grafts	Socket preservation procedures	Socket Preservation adopting deproteinized bovine bone mineral (DBBM) did not provide extra benefit regard to post-extraction new bone generation compared to natural healing.

**Table 2:** Excluded articles.

Authors	Reference	Year	Reason for Exclusion
Castro et al.	[49]	2017	no histological results
Del Fabbro et al.	[50]	2014	full text not found
Fawzy El-Sayed et al.	[51]	2019	different topic
Helgeland et al.	[52]	2018	different topic
Granate-Marques et al.	[53]	2019	different topic and comorbidity
Monje et al.	[54]	2016	different topic
Ragucci et al.	[55]	2019	different topic
Reynolds and Aichelmann-Reidy	[56]	2012	no systematic review
Schliephake et al.	[57]	2018	no histologic results
